# Characterization of Pneumococcal Colonization Dynamics and Antimicrobial Resistance Using Shotgun Metagenomic Sequencing in Intensively Sampled South African Infants

**DOI:** 10.3389/fpubh.2020.543898

**Published:** 2020-09-22

**Authors:** Rendani I. Manenzhe, Felix S. Dube, Meredith Wright, Katie Lennard, Stephanie Mounaud, Stephanie W. Lo, Heather J. Zar, William C. Nierman, Mark P. Nicol, Clinton Moodley

**Affiliations:** ^1^Division of Medical Microbiology, Faculty of Health Sciences, University of Cape Town, Cape Town, South Africa; ^2^Department of Molecular and Cell Biology, Faculty of Science, University of Cape Town, Cape Town, South Africa; ^3^J. Craig Venter Institute, Rockville, MD, United States; ^4^Division of Computational Biology, Faculty of Health Sciences, University of Cape Town, Cape Town, South Africa; ^5^Parasites and Microbes Program, The Wellcome Sanger Institute, Cambridge, United Kingdom; ^6^Department of Paediatrics and Child Health, Red Cross War Memorial Children's Hospital and South African - Medical Research Council Unit on Child & Adolescent Health, University of Cape Town, Cape Town, South Africa; ^7^Division of Infection and Immunity, University of Western Australia, Perth, WA, Australia; ^8^National Health Laboratory Service, Groote Schuur Hospital, Cape Town, South Africa

**Keywords:** nasopharyngeal, *Streptococcus pneumoniae*, pneumococcal conjugate vaccine, serotypes, shotgun metagenomic sequencing, resistance determinants, multi locus sequence typing

## Abstract

**Background:** There remains a significant proportion of deaths due to pneumococcal pneumonia in infants from low- and middle-income countries despite the marginal global declines recorded in the past decade. Monitoring changes in pneumococcal carriage is key to understanding vaccination-induced shifts in the ecology of carriage, patterns of antimicrobial resistance, and impact on health. We longitudinally investigated pneumococcal carriage dynamics in PCV-13 vaccinated infants by collecting nasopharyngeal (NP) samples at 2-weekly intervals from birth through the first year of life from 137 infants. As a proof of concept, 196 NP samples were retrieved from a subset of 23 infants to explore strain-level pneumococcal colonization patterns and associated antimicrobial-resistance determinants. These were selected on the basis of changes in serotype and antibiogram over time. NP samples underwent short-term enrichment for streptococci prior to total nucleic acid extraction and whole metagenome shotgun sequencing (WMGS). Reads were assembled and aligned to pneumococcal reference genomes for the extraction of pneumococcal and non-pneumococcal bacterial reads. Pneumococcal contigs were aligned to the Antibiotic Resistance Gene-ANNOTation database of acquired AMR genes. *In silico* pneumococcal capsular and multilocus sequence typing were performed.

**Results:** Of the 196 samples sequenced, 174 had corresponding positive cultures for pneumococci, of which, 152 were assigned an *in silico* serotype. Metagenomic sequencing detected a single pneumococcal serotype in 85% (129/152), and co-colonization in 15% (23/152) of the samples. Twenty-two different pneumococcal serotypes were identified, with 15B/15C and 16F being the most common non-PCV13 serotypes, while 23F and 19A were the most common PCV13 serotypes. Twenty-six different sequence types (STs), including four novel STs were identified *in silico*. Mutations in the *fol*A and *fol*P genes, associated with cotrimoxazole resistance, were detected in 89% (87/98) of cotrimoxazole-non-susceptible pneumococci, as well as in the *pbp*1a and *pbp*2x genes, in penicillin non-susceptible ST7052^15B/15C^ isolates.

**Conclusions:** Metagenomic sequencing of NP samples is a valuable culture-independent technique for a detailed evaluation of the pneumococcal component and resistome of the NP microbiome. This method allowed for the detection of novel STs, as well as co-colonization, with a predominance of non-PCV13 serotypes in this cohort. Forty-eight resistance genes, as well as mutations associated with resistance were detected, but the correlation with phenotypic non-susceptibility was lower than expected.

## Introduction

*Streptococcus pneumoniae* (the pneumococcus) is a frequent bacterial cause of infections such as bacterial pneumonia, otitis media, meningitis, sinusitis, and bacteraemia in young children, and is a major cause of morbidity and mortality (1–4). Globally, pneumococcal pneumonia was responsible for an estimated 393,000 deaths in children <5 years of age in 2015 (5). Asymptomatic pneumococcal nasopharyngeal (NP) carriage is common among infants, a reservoir for transmission and precedes the development of infections (6). Pneumococci are classified into nearly 100 serotypes, based on the antigenic specificity of the polysaccharide capsule (7, 8). Children are often sequentially colonized by multiple different serotypes (9, 10), and may be co-colonized by different pneumococcal serotypes at the same time (11).

Immunization with the pneumococcal conjugate vaccine (PCV) has substantially reduced NP carriage and invasive pneumococcal disease caused by serotypes represented in the vaccine (12, 13). In addition, the introduction of PCV has resulted in a reduction in antimicrobial resistant pneumococci amongst circulating strains due to the inclusion of serotypes associated with high antibiotic resistance in the conjugate vaccine (14). However, non-vaccine-serotypes have emerged among both carriage and disease-causing isolates, and are increasingly associated with antimicrobial resistance (15, 16). Increasing pneumococcal resistance to different classes of antibiotics, including beta-lactams, macrolides, tetracyclines, and cotrimoxazole, has compromised the effectiveness of antibiotics to treat pneumococcal infections (17).

Pneumococci are most commonly identified from carriage and disease samples using bacterial culture followed by phenotypic and genotypic characterization of the isolates (18, 19). The Quellung method, based on antisera reactions, is currently the gold standard for pneumococcal serotyping, but requires viable isolates in pure culture. To detect colonization with multiple serotypes, typing of many individual (often identical) pneumococcal colonies from each NP sample is required (20). Microarrays are able to detect co-colonization by all known pneumococcal serotypes, and depending on the microarray panel used, and targets included, may provide additional information on virulence and resistance determinants present. This method is however limited and cannot provide information on genes not included in the array (21, 22). Sequential multiplex PCR assays are increasingly used for serotyping, and can be done directly on the NP sample, however, this is relatively laborious, costly, and limited to serotypes targeted by the PCR (23). None of these methods are able to provide a more detailed, strain-level characterization, important for pneumococci, as capsular switching often results in different serotypes within the same lineage (24).

Metagenomic approaches, where the collective genomes of all organisms recovered directly from a sample are sequenced, is a high-throughput approach to investigate the members of a microbial community in an ecological niche, and represents an alternative to culture-dependant methods for microbial characterization (25). In this study, we explored the use of shotgun metagenomic sequencing to characterize the pneumococcal component of the NP, including identification of co-colonization with multiple serotypes and antimicrobial resistance, among PCV-vaccinated children participating in a South African birth cohort.

## Materials and Methods

### Study Population and Sampling

Children were enrolled in a longitudinal birth-cohort study, the Drakenstein Child Health Study (26), in the Western Cape Province of South Africa. NP swabs were collected every second week from birth through the first year of life, from 137 infants during 2012–2013 (10). Infants received 2+1 doses of 13-valent pneumococcal conjugate vaccine (PCV13) at 6 weeks, 14 weeks, and 9 months according to the national immunization program in South Africa. Details of the study population and sampling have been previously described (26). The study was approved by the University of Cape Town, Faculty of Health Sciences Human Research Ethics Committee (Reference numbers 401/2009 and 235/2016). Written informed consent was obtained from mothers.

### Identification and Antimicrobial Characterization of Pneumococci

The collected NP swabs were immediately placed into 1 ml skim milk-tryptone-glucose-glycerol (STGG) medium, transported on ice within 2 h of collection, and stored at −80°C for batch processing within a week. A 10 μl aliquot of the NP-STGG was inoculated onto a blood agar plate (Oxoid Columbia base with 5% sheep blood) containing gentamicin (5 μg/ml) and incubated overnight at 37°C, in 5% CO_2_. Presumptive pneumococcal isolates were identified as previously described (27); only a single pneumococcal isolate was selected for conventional characterization from each sample. All serotypes were initially deduced by sequetyping and subsequently confirmed by Quellung, as previously described (28, 29).

Susceptibility to penicillin (assessed using oxacillin disc), erythromycin, and cotrimoxazole were assessed using the disc diffusion method, and interpreted using Clinical and Laboratory Standards Institute (CLSI) guidelines (30). Penicillin minimum inhibitory concentrations (MICs) were determined using the E-test method (bioMérieux, Marcy I'Etoile, France), according to the manufacturer's instructions. Intermediate and resistant pneumococcal isolates were collectively regarded as non-susceptible.

### Broth Enrichment of NP Samples for Pneumococci

In order to explore the utility of metagenomic sequencing to detect colonization with multiple serotypes and identify antimicrobial-resistance determinants in this exploratory study, a total of 196 NP-STGG samples were purposively selected from a subset of 23 of the 137 infants described above. These samples were selected based on changes in pneumococcal serotype and antibiogram over time identified using phenotypic methods. For the purpose of comparison and longitudinally assessing colonization dynamics with other potential pathogens, 22 out of 196 samples from a subset of 23 infants which were culture negative for *S. pneumoniae*, were also included. The NP-STGG samples were enriched as previously described, with minor modifications (31). Briefly, 200 μl of an NP-STGG sample was transferred to 6 ml Todd-Hewitt Broth (without antibiotics), containing 0.5% yeast extract and 17% fetal bovine serum. The liquid culture was incubated at 37°C with 5% CO_2_, without shaking for 6 h. The culture was then centrifuged at 9,000 rpm for 10 min at 4°C. Total nucleic acid extraction was performed on the collected pellet using the QIAsymphony SP automated platform (Qiagen, Hilden, Germany) with the QIAsymphony Virus/Bacteria Mini Kit (Cat. No. 931036) following the manufacturer's instructions. Nucleic acid concentrations and purity were determined using the NanoDrop® ND-100 (Thermo Fishers Scientific, Waltham, USA). The purified nucleic acid was stored at −20°C and sequenced within a month.

### Metagenomic DNA Sequencing, Assembly and *in silico* Typing

Total nucleic acid was subjected to shotgun sequencing on the MiSeq platform using the MiSeq Reagent Kit v3 (600-cycle) (Illumina, San Diego, USA) at the J. Craig Venter Institute, Rockville, USA. Metagenomic DNA sequencing protocols and the pipeline used to assemble the reads and evaluate the assembly have been previously described (31). Reads were assembled using metaSPAdes (32), and aligned to the created serotype nucleotide database using BLASTn, with an identity over 98% considered a match (31). In addition, assembly-based *in silico* multi-locus sequence typing (MLST) was performed using LOCUST as previously described (33, 34).

Serotypes identified by Quellung/sequetyping (29), were compared to *in silico* serotypes identified from metagenomic sequences. Culture-based and metagenomic-assigned serotypes were considered concordant if the serotype detected by Quellung/sequetyping was detected by shotgun sequencing (either as a single serotype or amongst co-colonizing serotypes detected).

### Phylogenetic Tree Construction

A phylogenetic tree of pneumococcal genomes was constructed to assess strain relatedness. Assembly-based (metaSPAdes assembler) variant calling (32), was performed using The Northern Arizona Single Nucleotide Polymorphism Pipeline (NASP) (35). All completed pneumococcal genomes available on the NCBI database were included in the NASP run and the *S. pneumoniae* R6 genome (Accession number AE007317) was used as reference genome. The aligned base calls for each of the core variant positions identified by NASP were used for the construction of the maximum likelihood tree (36).

### Analysis of Genetic Determinants of Antimicrobial-Resistance

Pneumococcal contigs (contigs that mapped to reference pneumococcal genome R6) were compared to the Antibiotic Resistance Gene-Annotation (ARG-ANNOT) database by BLAST alignment (37). Results were filtered using ≥90% sequence identity over 80% of the sequence length of the reference antibiotic-resistance gene as cut-offs. The *pbp, fol*A [encoding dihydropteroate synthase (DHPS)], and *fol*P [encoding dihydrofolate reductase (DHFR)] gene mutations associated with resistance to beta-lactams, trimethoprim, and sulfonamides, respectively, were investigated by manual local alignment, as the ARG-ANNOT database does not automatically detect these mutations. The ARG-ANNOT database only included the *pbp*1a (JN645776) and *pbp*1b (AF101781) genes of wild-type pneumococcal strains. Therefore, local alignments of the *pbp*1a (JN645776.1), *pbp*2x (JN645706.1), *pbp*2b (DQ056780.1), *fol*A_R6 (Gene ID 4442919), and *fol*P_R6 (Gene ID 4443057) were performed using BLASTn (80% identity and 60% gene length coverage).

For each individual, and for each of the genes, sequences from the longitudinal samples were aligned with Multiple Alignment using Fast Fourier Transform (MAFFT) (38). Aligned sequences were viewed and translated to amino acids in AliViewer (version 1.18.1) for active site mutation analysis. The transpeptidase domains of the *pbp*1a, 2x, and 2b, from the wild type *S. pneumoniae* R6 strain were used as references. Phylogenetic trees were constructed with Molecular Evolutionary Genetic Analysis software (MEGA version 7.0.26) using a neighbor-joining method and bootstrapping 1000 replicates. *pbp*1a (JN645776.1), *pbp*2x (JN645706.1), and *pbp*2b (DQ056780.1) were used as reference gene sequences in the construction of the phylogenetic trees.

## Results

### Participant Characteristics

**Figure 1:**
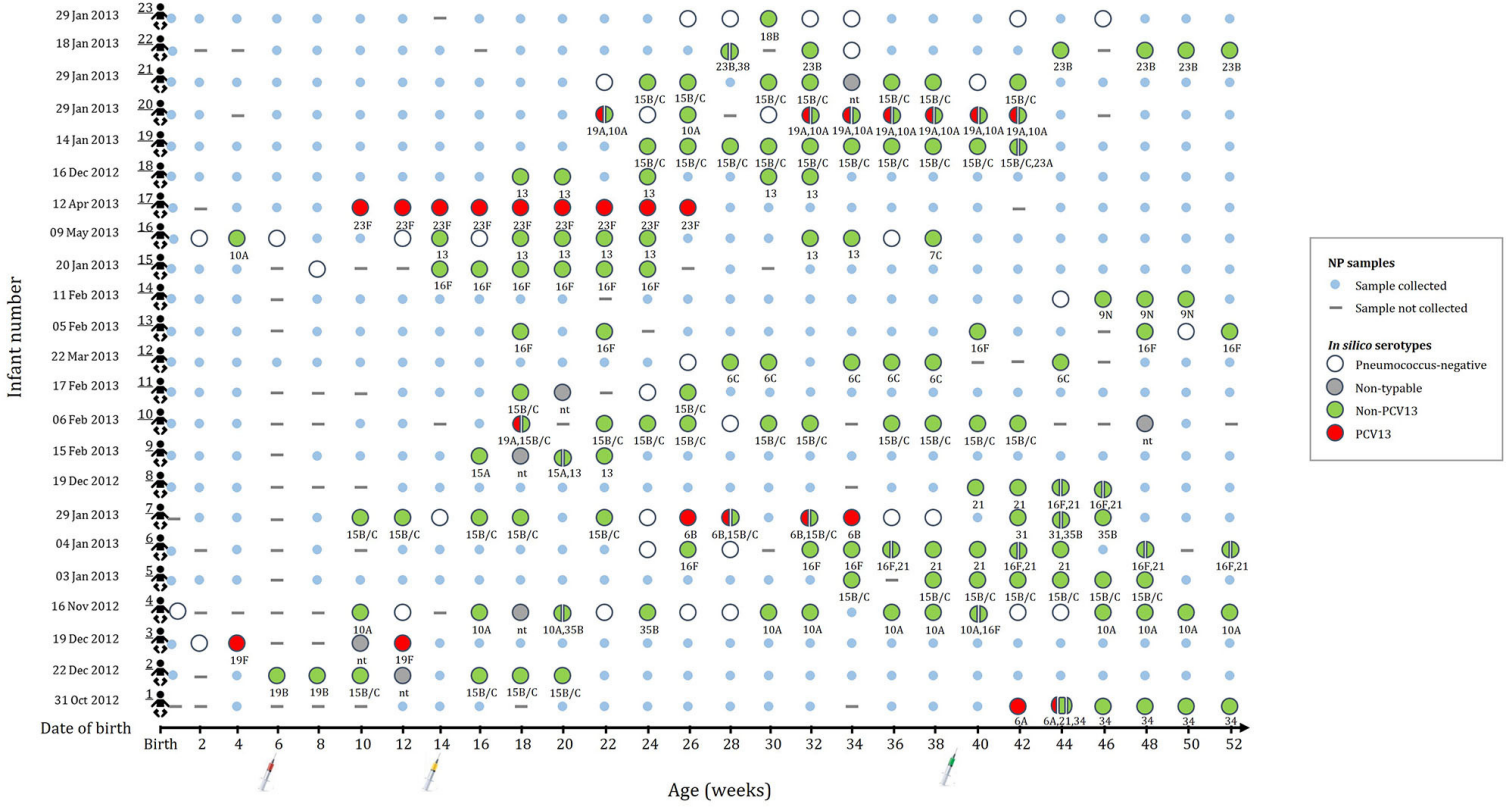
This figure indicates the number of samples selected for metagenomic sequencing and serotype assignments for the 196 nasopharyngeal (NP) samples selected from 23 infants (shown in rows 1 to 23). None of the infants were colonized until 4 weeks of age. Small blue circles represent the NP samples that were collected but not included for shotgun metagenomic sequencing. Large circles (colors represent serotype group) represent the collected NP samples selected for shotgun metagenomic sequencing. Split circles represent samples with co-colonization with multiple serotypes. *in silico* serotypes are displayed for samples selected for shotgun metagenomic sequencing. (-) NP sample not collected. (nt) Non-typeable.

A subset of 196 NP samples from 23 infants was selected for shotgun sequencing to investigate the pneumococcal population structure. The number of NP samples selected for sequencing ranged from 4 to 21 samples per infant (average of 8.5 samples); selected samples and age at sampling (average 14.9 weeks) for each of the infants is shown in Figure 1. Thirteen of the infants were males (57%). The mean birth weight was 3.0 kg (range, 2.4–3.8 kg) with only one preterm infant. Eight infants were HIV exposed (born to HIV-infected mothers), but were HIV-uninfected.

### Metagenomic Sequencing Results

The average number of reads per sample was 13 million (ranging from 80 thousand to 93 million reads per sample) despite low input DNA concentrations (data not shown). The average number of microbial reads per sample that mapped to the R6 pneumococcal reference genome was 4.5 million (range, 141 reads−32 million reads per sample) with an average coverage of 308.53X (range, 0.01X−2239.71X).

### *In silico* Serotypes and Sequence Types

Of the 196 samples sequenced, 174 had a corresponding positive pneumococcal culture. Shotgun sequencing detected pneumococcal reads in all 174 samples, however, 15 samples had no reads covering the required genomic regions (cps, housekeeping genes, and core variant positions identified by the NASP) of the pneumococcal genomes and were therefore excluded from further analyses. *In silico* serotypes using shotgun sequencing were assigned in 96% (152/159) of the remaining samples and serotypes were assigned in 93% (148/159) of samples by both Quellung/sequetyping and shotgun sequencing. The concordance between Quellung/sequetyping and shotgun *in silico* typing was 86% (127/148) (Supplementary Table 1). Co-colonization with two (*n* = 22) or three (*n* = 1) different serotypes was detected in 15% (23/152) of the samples from ten infants using shotgun sequencing. The bioinformatic analyses of these 23 samples produced highly reliable and reproducible alignment results with high read mapping counts, negating the possibility of mosaic loci from other *Streptococcus* species. Since Quellung/sequetyping typing was only performed on a single colony per sample, co-colonization was not detected using this method.

**Figure 2:**
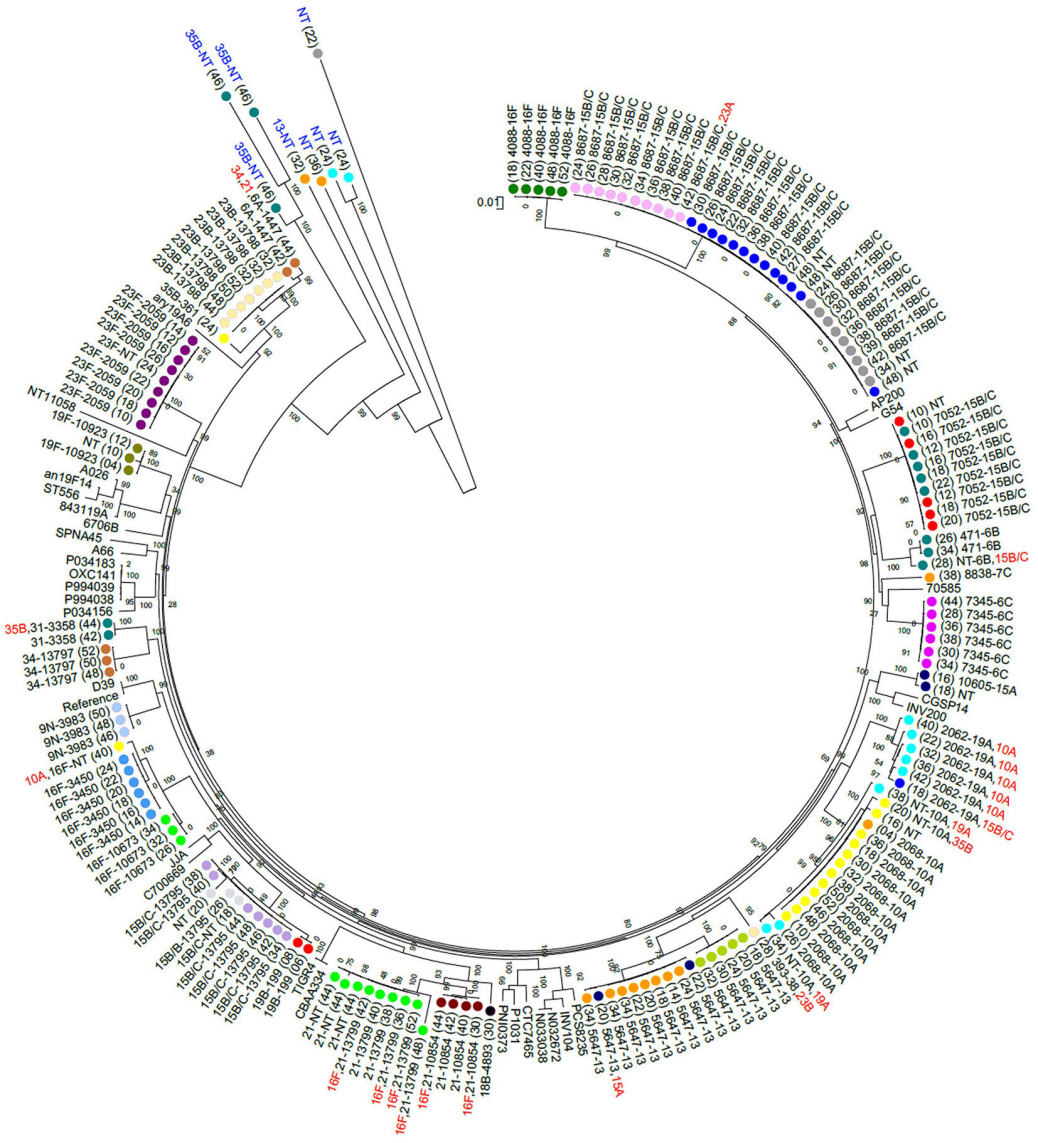
A maximum likelihood whole genome phylogenetic tree of pneumococcal isolates recovered from 23 infants. Bootstrap values are shown on each branch and the scale bar represents the number of SNPs. Circles with the same color represent longitudinal samples from the same infant. Numbers in brackets indicate the age in weeks at each time-point. The sequence type (ST) detected in each sample is shown as a number, followed by the associated serotype. Serotypes indicated in red were among co-colonizing strains and had genomes with lower coverage than the other co-detected strain. Five out of 15 samples that had low reads mapping to pneumococcal genomes are indicated in blue text. NT, Non-typeable. Completed pneumococcal genomes available on the NCBI database were included and the R6 genome was used as a reference. The samples clustered according to ST but not serotype. Persistent colonization with the same genotype was common.

Non-PCV13 serotypes were more commonly detected than PCV13 serotypes (Figure 1). Twenty-two different pneumococcal serotypes were identified *in silico*, with 15B/15C (*n* = 49), 16F (*n* = 21), 10A (n = 21), 13 (*n* = 14), and 21 (*n* = 12) being the most common non-PCV13 serotypes, and 23F (*n* = 9), and 19A (*n* = 8) being the most common PCV13 serotypes. Serotypes and STs identified are shown in Supplementary Table 2. MLST was assigned in 89% (142/159) of the samples representing 26 different MLST profiles (Figure 2). Eleven STs (non-PCV13-related: ST8687^15B/15C^, ST5647^13^, ST7345^6C^, ST4088^16F^, ST10854^21^, ST10673^16F^, ST10605^15A^, and ST8838^7C^; and PCV13-related: ST2059^23F^, ST2062^19A^, and ST10823^19F^) have previously been described only in South Africa, while five STs associated with non-PCV13 serotypes (ST2068^10A^, ST3450^16F^, ST3358^31^, ST199^19B^, and ST393^38^) had not been previously described in Africa. In addition, four novel STs (ST13795^15B/15C^, ST13797^34^, ST13798^23B^, and ST13799^21^) were identified (Figure 2 and Supplementary Table 2).

### Antimicrobial-Resistance Determinants

Phenotypic antibiotic susceptibility profiles were determined for the 159 pneumococcal isolates obtained from samples included for this analysis (39). Overall, 18% (29/159), 17% (28/159), and 61% (98/159) of the isolates were non-susceptible to penicillin, erythromycin, and cotrimoxazole, respectively.

**Figure 3:**
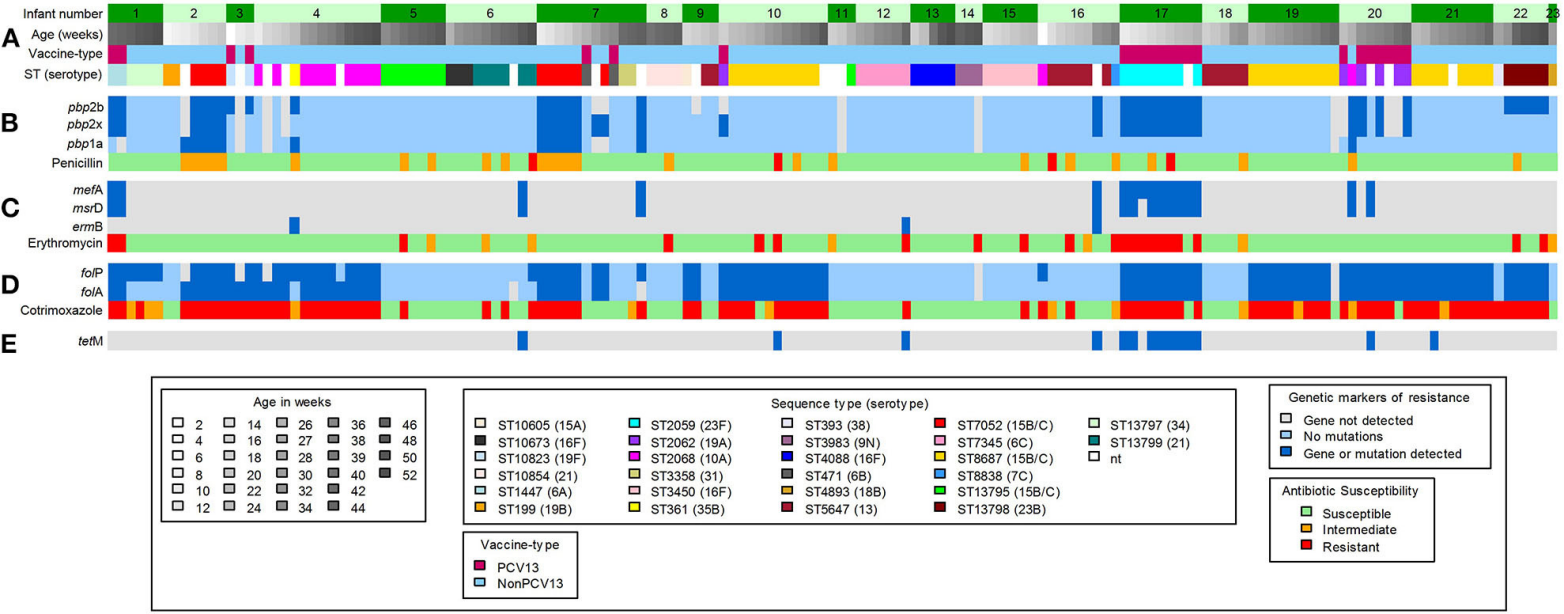
Shotgun sequence-derived molecular sequence type and resistome (from direct sequencing of NP swabs), and phenotypic antimicrobial susceptibility results (from pneumococcal isolates) in 159 out of 196 NP samples obtained from 23 infants (assigned 1 to 23, top row). **(A)** *in silico* sequence types (STs) and associated serotypes detected in longitudinal NP samples from each infant. **(B)** *pbp* gene mutations and associated penicillin phenotypic susceptibility profiles; light blue color indicates wild-type *pbp* genes; the dark blue color indicates detected mutations within or close to the conserved motifs for the pbp genes. **(C)** Macrolide-resistance genes and associated erythromycin phenotypic susceptibility profiles; the dark blue color indicates detected resistance genes. **(D)** *fol*A and *fol*P gene mutations and associated cotrimoxazole phenotypic susceptibility profiles; light blue color indicates wild-type *fol*A and *fol*P genes; dark blue color indicates detected mutations, in *fol*A and *fol*P, that reduce the affinity of trimethoprim or sulfamethoxazole. **(E)** Detected tetracycline resistance gene; dark blue color indicates detected resistance gene. Gray color in **(B–E)** indicates that no genes were detected.

Using *in silico* analyses, a total of 48 acquired antimicrobial-resistance (AMR) genes were identified from pneumococcal contigs in 20 samples collected from 10 infants (Figure 3). The AMR genes detected included macrolide-lincosamide-streptogramin B resistance (MLS_B_) (*msr*D, *mef* A and *erm*B), and tetracycline resistance (*tet*M) genes (Figure 3). The average sequence length coverage across the reference genes was 99% (range, 81–100%) while the average sequence depth was 160X (range, 1–411X).

Phenotypic non-susceptibility to erythromycin was detected in 18% (28/159) isolates (Figure 3). Among the 28 samples from which these isolates were obtained, genes predicted to confer macrolide non-susceptibility were detected in 39% (11/28) samples [*msr*D and *mef* A (*n* = 9), *mef* A (*n* = 1), and *erm*B (*n* = 1)] from three infants. In addition, macrolide resistance genes were detected in a further six samples, from five infants, from which erythromycin-non-susceptible pneumococci were not cultured (Figure 3); two of these samples had co-colonization with multiple serotypes.

Pneumococcal *pbp* genes were analyzed for mutations associated with beta-lactam resistance. No indels were identified in any of the extracted *pbp* gene sequences, but multiple amino acid changes were identified at various positions within the transpeptidase domains. Concordance between penicillin non-susceptibility and the presence of resistance conferring mutations was low (52%, 15/29). Interestingly, resistance conferring mutations were also identified among 14 penicillin susceptible strains (Figure 3). Pneumococcal strains with penicillin MICs between 0.064 and 8.0 μg/ml commonly carried mutations within the transpeptidase domains of the *pbp*1a and *pbp*2b genes, but not in *pbp*2x (Supplementary Figure 1), compared to the wild-type pneumococcal strain R6. *pbp* point mutations known to contribute to beta-lactam resistance are shown in Supplementary Table 3. The S351A and P432T point mutations which are within or close to the conserved motifs of the *pbp*1a gene were identified in penicillin-non-susceptible ST7052^15B/15C^ (*n* = 9), ST361^35B^ (*n* = 1), and ST2068^10A^ (*n* = 1) isolates from four infants (Figure 3 and Supplementary Table 3). Amino acid substitution H394L, in the *pbp*2x gene, was only identified among PCV13 serotypes 23F (*n* = 9), 19A (*n* = 4), and 6A (*n* = 2), from four infants, irrespective of penicillin susceptibility (Supplementary Table 4). These serotypes (23F, 19A, and 6A) had identical amino acid sequences of the transpeptidase domain of the *pbp*2x gene despite having different STs (Supplementary Figure 3). Pneumococcal strains identified in this study had a higher divergence in the transpeptidase domain sequences of the *pbp*1a and *pbp*2x genes than in the *pbp*2b gene (Supplementary Figures 2–4).

Ninety-eight out of 159 (61%) isolates were phenotypically non-susceptible to cotrimoxazole (Figure 3). The *fol*A (I100L) and *fol*P (6-bp insertion in the region encoding amino acid 58 to 67) gene mutations, known to confer resistance to cotrimoxazole (40, 41), were detected in 89% (87/98) of the samples from which the non-susceptible isolates were obtained (Figure 3 and Supplementary Table 4). Combinations of resistance mutations including I100L plus R_59_P_60_ (*n* = 45), and I100L plus S_62_Y_63_ (*n* = 24) were commonly detected (Supplementary Table 4).

## Discussion

This study explored the use of shotgun metagenomic sequencing as an alternative approach to culture-based testing to investigate pneumococcal NP colonization and associated antimicrobial-resistance determinants, in a South African birth cohort. We were able to derive pneumococcal serotypes and sequence types, and to identify co-colonization and antimicrobial-resistance determinants directly from shotgun sequence data. Since NP samples from apparently healthy individuals generally have low numbers of bacterial cells, we used short-term broth enrichment, which has previously been shown to successfully enrich streptococci (31). Since antibiotics may select for resistant strains, the current study used broth enrichment without antibiotics to encourage growth of all *S. pneumoniae* present without selective pressure (42).

There was complete concordance between detection of pneumococcal sequences and positive culture for pneumococci. However, of the 174 culture positive samples sequenced, 15 samples produced poor sequence read mapping to the reference pneumococcal genomes, despite good overall read counts. These 15 samples were excluded from further analyses as they were likely other *Streptococcus* species, which would have been enriched in culture, but map poorly to the reference sequences used, despite having high read counts. There was good correlation (86%) between conventional typing methods and shotgun sequencing in assigning pneumococcal serotypes. Discordant serotype results were predominantly from samples where only one serotype was identified by shotgun sequencing, and were therefore not due to detectable co-colonization. Both Quellung and sequetyping are not infallible in assigning pneumococcal serotypes (23). Discordant serotypes between conventional and *in silico* typing were likely due to the increased resolution on shotgun sequencing over conventional methods, which may be less specific (43). Shotgun metagenomic sequencing produced robust serotyping results, based on sequence coverage and depth, but this technique is, at present, relatively expensive, time consuming, and computationally intensive for routine typing (44). Whole-genome sequencing of cultured pneumococcal isolates produces reliable serotyping results and can generate additional genetic information, but is typically performed on single isolates, and may thus fail to detect co-colonization, or exclude samples with non-viable bacteria (45). On the other hand, microarray techniques which may or may not require a culture-enrichment step have the potential to detect multiple serotypes within a sample, however, this technique can only detect serotypes and other genetic elements included in the array, and can be technically challenging to distinguish closely related serotypes (46).

Eleven percent (17/159) of the samples produced good alignments of most alleles, but could not be assigned a multilocus sequence type due to a lack of resolution at all the loci necessary for typing. This was observed in four out of 23 samples where co-colonization with multiple serotypes was detected, and in samples with low estimated sequencing coverage (31). Bioinformatic analyses indicated this was due to low read depth or coverage of certain alleles, primarily at the terminal nucleotide positions which are needed for assigning a locus identity. In samples where sequence types were assigned, we observed a strong association between certain pneumococcal serotypes and multilocus sequence types. However, serotypes 15B/15C (ST7052, ST8687, and ST13795) and 16F (ST4088 and ST3450/ST10673) were associated with multiple STs. Serotypes 15B/15C and 16F were the predominant serotypes detected in our cohort, and highly prevalent serotypes tend to be more diverse (47). No other ST was associated with more than one serotype, in line with previous observations (48).

Shotgun sequencing detected co-colonization with multiple serotypes in 15% of the samples; these would have been difficult or laborious to detect using the Quellung method, since multiple colonies would have to be tested individually, or pooled colonies tested with multiple reagents (45). This rate of co-colonization was lower than that reported in Malawi among children aged 0–13 years (40%, 46/116) (49), but comparable to that reported among children <2 years of age in Tehran (17%, 225/1302) (50). In the current study, serotype 19A isolates (from two infants) were only detected using shotgun sequencing (and not culture), in samples with multiple serotypes. Detection of circulating serotype 19A is important in epidemiological studies, since 19A is included in the PCV13 administered to infants. Culture-dependent techniques are biased to detect the most abundant serotype in a sample and are likely to miss co-colonization with less abundant serotypes (51). Carriage of multiple pneumococcal serotypes is also important since it provides an opportunity for horizontal gene transfer, which is one of the most common mechanisms driving pneumococcal evolution (52).

Eleven STs identified in this study, including ST2059^23F^, ST2062^19A^, and ST10823^19F^, matched other STs which have only been described among isolates from South Africa, and may therefore be endemic. These strains are associated with serotypes included in the PCV13, which is currently administered in the South African schedule, and which all infants in this study received. Five STs identified here are reported for the first time in an African country. Additionally, four novel STs were identified, namely: ST13795^15B/15C^, ST13797^34^, ST13798^23B^, and ST13799^21^.

The AMR genes identified by pneumococcal resistome analysis were *msr*D, *mef* A, *erm*B, and *tet*M. Macrolide-resistance genes (*msr*D, *mef* A, or *erm*B), predicted to confer resistance, were detected in 11/28 samples with erythromycin-non-susceptible pneumococci. Acquired AMR genes were not detected in the remaining samples, and the resistance observed could be due to other mechanisms of macrolide resistance not investigated here (53). The frequency of *msr*D or *mef* A was higher than that of *erm*B, and this is in contrast to what has been reported in other studies (54, 55). *Tet*M gene was detected in eight samples but this could not be associated with phenotypic resistance as isolates in this study were not tested for susceptibility to tetracycline, since it is not routinely used to treat pneumococcal infections.

A high proportion of cotrimoxazole-non-susceptibility (61%, 98/159) was observed in pneumococcal isolates present in samples included for shotgun sequencing. Cotrimoxazole, which is a combination of trimethoprim and sulfamethoxazole, inhibits folic acid biosynthesis, and non-susceptibility to this drug is conferred by the acquisition of mutations in *fol*A and *fol*P (40). The majority of cotrimoxazole-non-susceptible isolates (81%) in the current study possessed the I100L amino acid substitution in DHFR, and this mutation has been shown to be sufficient to confer high-level cotrimoxazole resistance (40, 41). The most common insertions detected in DHPS led to the duplication of R_58_P_59_ and S_62_Y_63_ in 56 and 27% of cotrimoxazole-non-susceptible isolates, respectively. These insertions have been shown to confer low-level cotrimoxazole resistance (40, 41). Most instances (71/86) of high-level cotrimoxazole resistance observed were due to both *fol*A I100L substitution and *fol*P insertion, and this has been previously described (56). Lack of associations in other isolates might be due to other mechanisms of resistance or loss of expression of the detected mutations (Supplementary Table 4) (56).

*Pbp* genes code for the penicillin-binding proteins (PBPs) which are essential for cell envelope bio-synthesis and are the target for beta-lactam antibiotics (57). Gene mutations occurring within or close to the *pbp*-conserved motifs within the transpeptidase domain are known to confer resistance to beta-lactams (58). Amino acid alterations in *pbp*1a, *pbp*2b, and *pbp*2x have been shown to be the most reliable markers for beta-lactam-resistance in pneumococci (59). A higher level of variation in the transpeptidase domain sequences of *pbp*1a and *pbp*2x, than in *pbp*2b was observed (Supplementary Figures 2–4). The *pbp*1a and *pbp*2x genes flank the capsule (*cps*) locus, which is prone to frequent recombination events (60), and recombination events involving the *cps* locus and one or both *pbp*1a and/or *pbp*2x genes have been observed (61). This could account for the higher level of variation observed in the *pbp*1a and *pbp*2x genes among the strains in this study.

In total, 29 out of 159 (18%) pneumococcal isolates were phenotypically non-susceptible to penicillin. Concordance between penicillin non-susceptibility and the presence of resistance conferring mutations was observed in 52% of the isolates. No known resistance conferring mutations were identified in the remaining penicillin non-susceptible strains, and the resistance observed may be due to the combination of other *pbp* and/or non-*pbp* mutations which may confer resistance in pneumococci (62). The presence of resistance conferring mutations among the 14 penicillin susceptible strains may indicate the poor predictability of these mutations for phenotypic resistance. Only P432T (in *pbp*1a) and T338P (in *pbp*2x) mutations were associated with phenotypic penicillin non-susceptibility in this study (Supplementary Table 3). The P432T and T338P mutations were detected in all nine penicillin-non-susceptible, ST7052^15B/15C^ isolates, from two infants. The P432T mutation, which is close to the ^428^SRN^430^ conserved motif (59), and the T338P mutation, occurring within the active ^337^STMK^340^ motif (63), decrease beta-lactam-binding affinity of PBP1a and PBP2x, respectively. The contribution of other mutations to penicillin non-susceptibility among our strains was unclear (Supplementary Table 3).

This study was limited by a small sample size and the use of metagenomic sequencing in samples obtained from a larger population is warranted. NP samples were enriched using broth culture without antibiotics which may have favored the growth of other bacteria over pneumococci, leading to low sequence reads counts and the inability to assemble the pneumococcal genomes in these samples.

## Conclusion

This study indicated the utility of direct metagenomic sequencing of NP samples to privide in-depth understanding of pneumococcal carriage and antimicrobial resistance determinants. There was complete concordance between culture and shotgun sequencing, with a high concordance between *in silico* and conventional serotyping, indicating a predominance of non-PCV13 serotypes in this cohort. This approach was also able to identify co-colonization. Serotypes endemic to South Africa, several not reported locally before, and four novel serotypes were also identified. There was however a poor correlation between phenotypic antimicrobial non-susceptibility and the detection of certain resistance determinants. This technique will contribute to understanding increasing vaccine failure rates, by providing stain-level data for vaccine design strategies which are effective in controlling carriage, preventing invasive disease, and limiting the potential spread of resistance.

## Data Availability Statement

The datasets presented in this study can be found in online repositories. The names of the repository/repositories: https://www.ebi.ac.uk/ena/browser/view/PRJEB37312.

## Ethics Statement

The studies involving human participants were reviewed and approved by Human Research Ethics Committee of the Faculty of Health Sciences, University of Cape Town and the Western Cape Provincial Child Health Research Committee. Written informed consent to participate in this study was provided by the participants' legal guardian/next of kin.

## Author Contributions

CM, FD, MN, HZ, and WN designed the study. RM, FD, CM, and SM performed data acquisition. RM, FD, CM, SL, MW, KL, and SM analyzed the data. RM, FD, and CM produced the first draft. All authors revised the work, approved the final version, and took accountability for all aspects of the work.

## Conflict of Interest

The authors declare that the research was conducted in the absence of any commercial or financial relationships that could be construed as a potential conflict of interest.
