# Supplementary material for: Characterization of Pneumococcal Colonization Dynamics and Antimicrobial Resistance Using Shotgun Metagenomic Sequencing in Intensively Sampled South African Infants
Source: Front Public Health. 2020 Sep 22;8:543898. doi: 10.3389/fpubh.2020.543898 (PMC7536305; doi:10.3389/fpubh.2020.543898)
Supplement: Supplementary file 1 [file Table_1.DOCX]

**Table S1:** Comparison between shotgun sequencing and conventional methods for pneumococcal serotyping

| No. of infants | Pneumococcal serotypes detected (no. of samples) | | Comment |
| --- | --- | --- | --- |
|  | Shotgun sequencing | Conventional methods |  |
| 3 | 13 (13) | 13 (11) | Concordant |
|  |  | 23A (1) | ˣ |
|  |  | 16F (1) | ˣ |
| 2 | 21 (5) | 21 (4) | Concordant |
|  |  | 16F (1) | ˣ |
| 1 | 31 (1) | 31 (1) | Concordant |
| 1 | 34 (4) | 34 (2) | Concordant |
|  |  | 21 (1) | ˣ |
|  |  | 16F (1) | ˣ |
| 3 | 10A (12) | 10A (11) | Concordant |
|  |  | 22F (1) | ˣ |
| 1 | 15A (1) | 15A (1) | Concordant |
| 7 | 15B/15C (45) | 15B/15C (42) | Concordant |
|  |  | 10A (1) | ˣ |
|  |  | 35B/C (1) | ˣ |
|  |  | nt (1) | ˣ |
| 3 | 16F (14) | 16F (11) | Concordant |
|  |  | 9V (2) | ˣ |
|  |  | 6C (1) | ˣ |
| 1 | 18B (1) | 18B (1) | Concordant |
| 1 | 19B (2) | 19B (2) | Concordant |
| 1 | 19F (2) | 19F (2) | Concordant |
| 1 | 23B (5) | 23B (3) | Concordant |
|  |  | 11A (1) | ˣ |
|  |  | nt (1) | ˣ |
| 1 | 23F (9) | 23F (1) | Concordant |
|  |  | 9V (6) | ˣ |
|  |  | 10A (1) | ˣ |
|  |  | 13 (1) | ˣ |
| 2 | 35B (2) | 35B (2) | Concordant |
| 1 | 6A (1) | 6A (1) | Concordant |
| 1 | 6B (2) | 6B (2) | Concordant |
| 1 | 6C (6) | 6C (6) | Concordant |
| 1 | 7C (1) | 7C (1) | Concordant |
| 1 | 9N (3) | 9N (3) | Concordant |
| 1 | 19A, 10A (7) | 10A (6) | Concordant |
|  |  | 19A (1) | Concordant |
| 1 | 6A, 21, 34 (1) | 6A (1) | Concordant |
| 1 | 10A, 35B (1) | 19C (1) | ˣ |
| 1 | 10A, 16F (1) | 16F (1) | Concordant |
| 2 | 21, 16F (6) | 21 (3) | Concordant |
|  |  | 16F (3) | Concordant |
| 1 | 6B, 15B/15C (2) | Nt (2) | ˣ |
| 1 | 31, 35B (1) | 35B (1) | Concordant |
| 1 | 13, 15A (1) | 13 (1) | Concordant |
| 1 | 15B/15C, 23A (1) | 23A (1) | Concordant |
| 1 | 23B, 38 (1) | 23B (1) | Concordant |
| 1 | 19A, 15B/15C (1) | 19A (1) | Concordant |
| 7 | nt (7) | * | ˣ |
| 12 | Negative^1^ (22) | Negative^2^ (22) | Concordant |

* 15B/15C (4), 10A (1), 19F (1), and 15A (1). ^1^ No pneumococcal reads detected, ^2^ Culture-negative for pneumococcus. (ˣ) Discordant results. Conventional and metagenomic-assigned serotypes were considered concordant if the serotype detected by conventional methods was among the co-colonizing serotypes.

**Table S2:** Distribution of *in silico* serotypes, associated STs, and MLST profiles.

| Serotype | Sequence type  (no. of samples) | No. of  infants | MLST allelic profile | | | | | | |  | Sequence type (other serotypes listed in the MLST database)^1^ |
| --- | --- | --- | --- | --- | --- | --- | --- | --- | --- | --- | --- |
|  |  |  | *aro*E | *gdh* | *gki* | *rec*P | *spi* | *xpt* | *ddl* |  |  |
| 15B/15C | ST8687 (28) | 3 | 1 | 32 | 97 | 5 | 10 | 88 | 18 |  | 6A |
|  | ST7052 (10) | 2 | 7 | 43 | 47 | 16 | 6 | 14 | 17 |  | none |
|  | ST13795* (8) | 2 | 2 | 13 | 14 | 1 | 17 | 4 | 14 |  | none |
| 13 | ST5647 (13) | 3 | 15 | 5 | 54 | 1 | 197 | 1 | 168 |  | none |
| 10A | ST2068 (12) | 3 | 2 | 40 | 4 | 19 | 10 | 1 | 27 |  | none |
| 23F | ST2059 (8) | 1 | 15 | 29 | 4 | 21 | 30 | 27 | 1 |  | 6A, 19A, 19F |
| 19A | ST2062 (6) | 2 | 1 | 5 | 53 | 32 | 14 | 20 | 199 |  | none |
| 16F | ST3450 (6) | 1 | 1 | 5 | 14 | 5 | 15 | 1 | 1 |  | none |
|  | ST4088 (5) | 1 | 50 | 8 | 1 | 16 | 6 | 88 | 14 |  | none |
|  | ST10673 (3) | 1 | 1 | 5 | 14 | 4 | 15 | 1 | 1 |  | none |
| 6C | ST7345 (6) | 1 | 1 | 10 | 9 | 43 | 15 | 1 | 17 |  | none |
| 21 | ST13799* (6) | 1 | 50 | 10 | 2 | 16 | 1 | 834 | 1 |  | none |
|  | ST10854 (4) | 1 | 8 | 10 | 2 | 138 | 1 | 26 | 1 |  | none |
| 23B | ST13798* (5) | 1 | 7 | 608 | 8 | 6 | 25 | 6 | 8 |  | none |
| 34 | ST13797* (4) | 1 | 12 | 5 | 54 | 38 | 27 | 14 | 18 |  | none |
| 9N | ST3983 (3) | 1 | 8 | 5 | 7 | 12 | 15 | 16 | 6 |  | 9V |
| 19B | ST199 (2) | 1 | 8 | 13 | 14 | 4 | 17 | 4 | 14 |  | 19A, 15B/15C, 3 |
| 6A | ST1447 (2) | 1 | 6 | 13 | 8 | 6 | 25 | 6 | 8 |  | 23F, 35B |
| 31 | ST3358 (2) | 1 | 1 | 2 | 29 | 1 | 43 | 14 | 8 |  | none |
| 19F | ST10823 (2) | 1 | 10 | 25 | 9 | 10 | 6 | 4 | 168 |  | none |
| 6B | ST471 (2) | 1 | 7 | 25 | 4 | 4 | 15 | 1 | 28 |  | 6A |
| 35B | ST361 (1) | 1 | 7 | 13 | 8 | 6 | 6 | 6 | 8 |  | 1, 23F, 6A, 6B, 19A, 23B |
| 38 | ST393 (1) | 1 | 10 | 43 | 41 | 18 | 13 | 49 | 6 |  | 25A, NT |
| 18B | ST4893 (1) | 1 | 2 | 5 | 2 | 16 | 1 | 26 | 1 |  | 18C |
| 7C | ST8838 (1) | 1 | 7 | 8 | 146 | 15 | 25 | 20 | 5 |  | none |
| 15A | ST10605 (1) | 1 | 2 | 18 | 6 | 10 | 17 | 12 | 19 |  | none |

^1^ Other serotypes associated with sequence types described in this study as listed in the PubMLST global database. * Novel STs. MLST profile could not be assigned for serotype 23A, which is not shown in the table.

**Table S3:** Deduced amino acid substitutions in penicillin-binding proteins (PBPs)

| Infant  no.^1^ | Sequence type | Serotype | Penicillin  phenotype | Amino acid substitution at position:^a^ | | | | | | | | | | | |
| --- | --- | --- | --- | --- | --- | --- | --- | --- | --- | --- | --- | --- | --- | --- | --- |
|  |  |  |  | PBP1a | | |  | PBP2x | | |  | PBP2b | | | |
|  |  |  |  | 351 | 371 | 432 |  | 338 | 394 | 546 |  | 451 | 614 | 624 | 629 |
| Ref | R 6 |  | S | S | T | P |  | T | H | L |  | T | L | A | A |
|  | NT | NT | I | A | . | T |  | X | X | X |  | X | X | X | X |
|  | ST7052 | 15B/15C | I | A | . | T |  | P | . | . |  | A | A | . | N |
| 2 | ST7052 | 15B/15C | I | A | . | T |  | P | . | . |  | A | A | . | N |
|  | ST7052 | 15B/15C | I | A | . | T |  | P | . | . |  | A | A | . | N |
|  | ST7052 | 15B/15C | I | A | . | T |  | P | . | . |  | A | A | . | N |
|  | ST7052 | 15B/15C | I | A | . | T |  | P | . | . |  | A | A | . | N |
|  | ST7052 | 15B/15C | I | A | . | T |  | P | . | . |  | A | A | . | N |
| 7 | ST7052 | 15B/15C | I | A | . | T |  | P | . | . |  | A | A | . | N |
|  | ST7052 | 15B/15C | I | A | . | T |  | P | . | . |  | A | A | . | N |
|  | ST7052 | 15B/15C | I | A | . | T |  | P | . | . |  | A | A | . | N |
|  | nt | 35B | S | A | A | T |  | A | . | V |  | A | T | G | . |
|  | nt | 15B/15C | S | X | X | X |  | P | . | . |  | X | X | X | X |
|  | ST7052 | 15B/15C | S | X | X | X |  | P | . | . |  | X | X | X | X |
| 4 | ST361 | 35B | I | A | . | T |  | . | . | . |  | A | S | . | E |
| 1 | ST1447 | 6A | S | . | . | . |  | . | L | . |  | A | S | . | E |
|  | ST1447 | 6A | S | X | X | X |  | . | L | . |  | A | S | . | E |
| 3 | ST10823 | 19F | S | . | . | . |  | . | . | . |  | A | . | . | . |
|  | ST10823 | 19F | S | . | . | . |  | . | . | . |  | A | . | . | . |
|  | ST2059 | 23F | S | . | . | . |  | . | L | . |  | A | . | . | . |
|  | ST2059 | 23F | S | . | . | . |  | . | L | . |  | A | . | . | . |
|  | ST2059 | 23F | S | . | . | . |  | . | L | . |  | A | . | . | . |
| 17 | ST2059 | 23F | I | . | . | . |  | . | L | . |  | A | . | . | . |
|  | ST2059 | 23F | S | . | . | . |  | . | L | . |  | A | . | . | . |
|  | ST2059 | 23F | R | . | . | . |  | . | L | . |  | A | . | . | . |
|  | ST2059 | 23F | S | . | . | . |  | . | L | . |  | A | . | . | . |
|  | nt | 23F | S | . | . | . |  | . | L | . |  | A | . | . | . |
|  | ST2059 | 23F | S | . | . | . |  | . | L | . |  | A | . | . | . |
| 10 | ST2062 | 19A | S | . | . | . |  | . | L | . |  | X | X | X | X |
| 20 | ST2068 | 10A | I | A | S | T |  | A | . | V |  | A | . | . | . |
|  | ST2062 | 19A | S | . | . | . |  | . | L | . |  | A | . | . | . |
|  | ST2062 | 19A | S | . | . | . |  | . | L | . |  | A | . | . | . |
|  | ST2062 | 19A | S | . | . | . |  | . | L | . |  | A | . | . | . |
| 22 | ST13798 | 23B | S | . | . | . |  | . | . | . |  | A | S | . | E |
|  | ST13798 | 23B | I | . | . | . |  | . | . | . |  | A | S | . | E |
|  | ST13798 | 23B | S | . | . | . |  | . | . | . |  | A | S | . | E |
|  | ST13798 | 23B | S | . | . | . |  | . | . | . |  | A | S | . | E |
|  | ST13798 | 23B | S | . | . | . |  | . | . | . |  | A | S | . | E |
| 16 | nt | 13 | S | . | . | . |  | A | . | V |  | A | T | G | . |

^1^ Assigned infant number. Only mutations at positions within or close to the conserved motifs in the transpeptidase domain in PBP1a (^370^STMK^373^, ^428^SRN^430^), PBP2x (^337^STMK^340^, ^395^SSN^397^, ^547^KSG^549^), and PBP2b (^448^SSN^450^, ^619^KTG^621^) are shown. ^a^ Identity with the amino acid from *S. pneumoniae* strain R6 is indicated by the dot. (X)- The gene was not detected. Pen- penicillin, Ery- erythromycin, Sxt- cotrimoxazole, NT- non-typable, S- Susceptible, I- Intermediate, R- Resistant.

**Table S4:** Amino acid variation of DHFR (*fol*A) and DHPS (*fol*P) associated with cotrimoxazole non-susceptibility

| Cotrimoxazole  phenotype  (no. of isolates) | No. of isolates (no. of infants) | Amino acid mutations | |
| --- | --- | --- | --- |
|  |  | *fol*A  substitution | *fol*P insertion |
| Resistant (n = 86) |  |  |  |
|  | 45 (8) | I100L | R_58_P_59_ |
|  | 24 (4) | I100L | S_62_Y_63_ |
|  | 2 (1) | I100L | S_61_S_62_ |
|  | 3 (3) | I100L | - |
|  | 1 (1) | I100L | wt |
|  | 2 (2) | wt | R_58_P_59_ |
|  | 1 (1) | wt | S_62_Y_63_ |
|  | 1 (1) | - | R_58_P_59_ |
|  | 7 (6) | wt | wt |
| Intermediate (n = 12) |  |  |  |
|  | 3 (2) | I100L | R_58_P_59_ |
|  | 1 (1) | I100L | S_62_Y_63_ |
|  | 4 (2) | wt | R_58_P_59_ |
|  | 4 (3) | wt | wt |
| Susceptible (n = 5) |  |  |  |
|  | 3* (2) | I100L | S_62_Y_63_ |
|  | 2 (2) | I100L | R_58_P_59_ |

Wt –Wild type gene, * Samples had co-colonization with multiple serotypes.


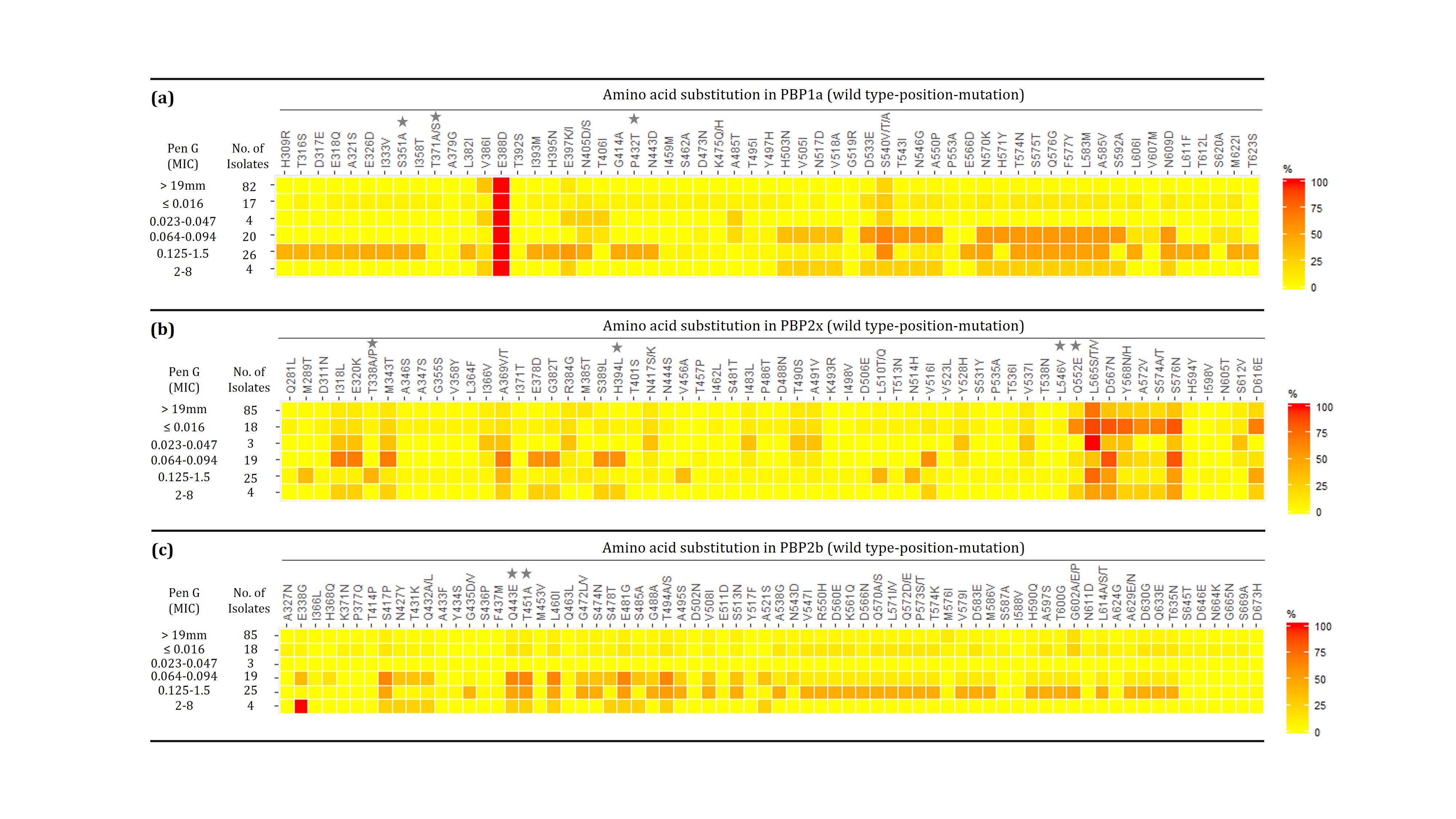


**Figure S1:** Codons with mutations within the transpeptidase domains of penicillin-binding proteins (PBPs). (a) PBP1a; (b) PBP2x; (c) PBP2b. Only amino acids that differ from the R6 wild-type strain (GenBank accession no. AE007317.1) are shown. Mutations at positions within or close to each of the conserved penicillin-binding motifs (^370^STMK^373^ and ^428^SRN^430^ in PBP1a, ^337^STMK^340^, ^395^SSN^397^, and ^547^KSG^549^ in PBP2x, and ^448^SSN^450^ in PBP2b) are indicated by the asterisks.


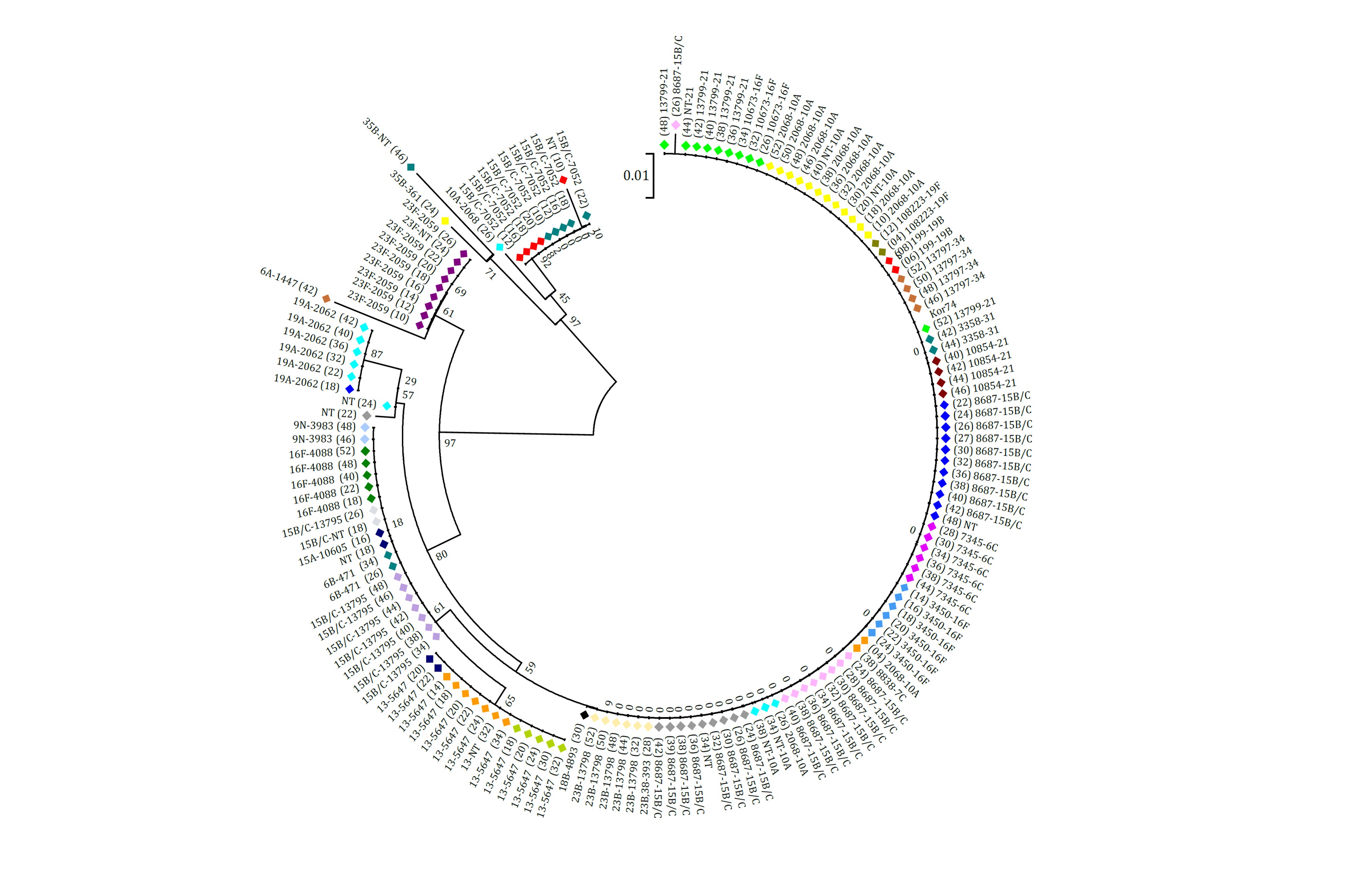


**Figure S2:** Phylogenetic tree, based on the transpeptidase domain of the *pbp*1a gene constructed, by MEGA using the neighbour-joining method. Bootstrap values (percentage) are based on 1,000 replications. The scale bar represents the genetic divergence as calculated by the MEGA software. Same colour represents the longitudinal samples from the same infant. The number in brackets indicate the age in weeks at each time-point. The ST detected in each sample is shown as a number, followed by the associated serotype.


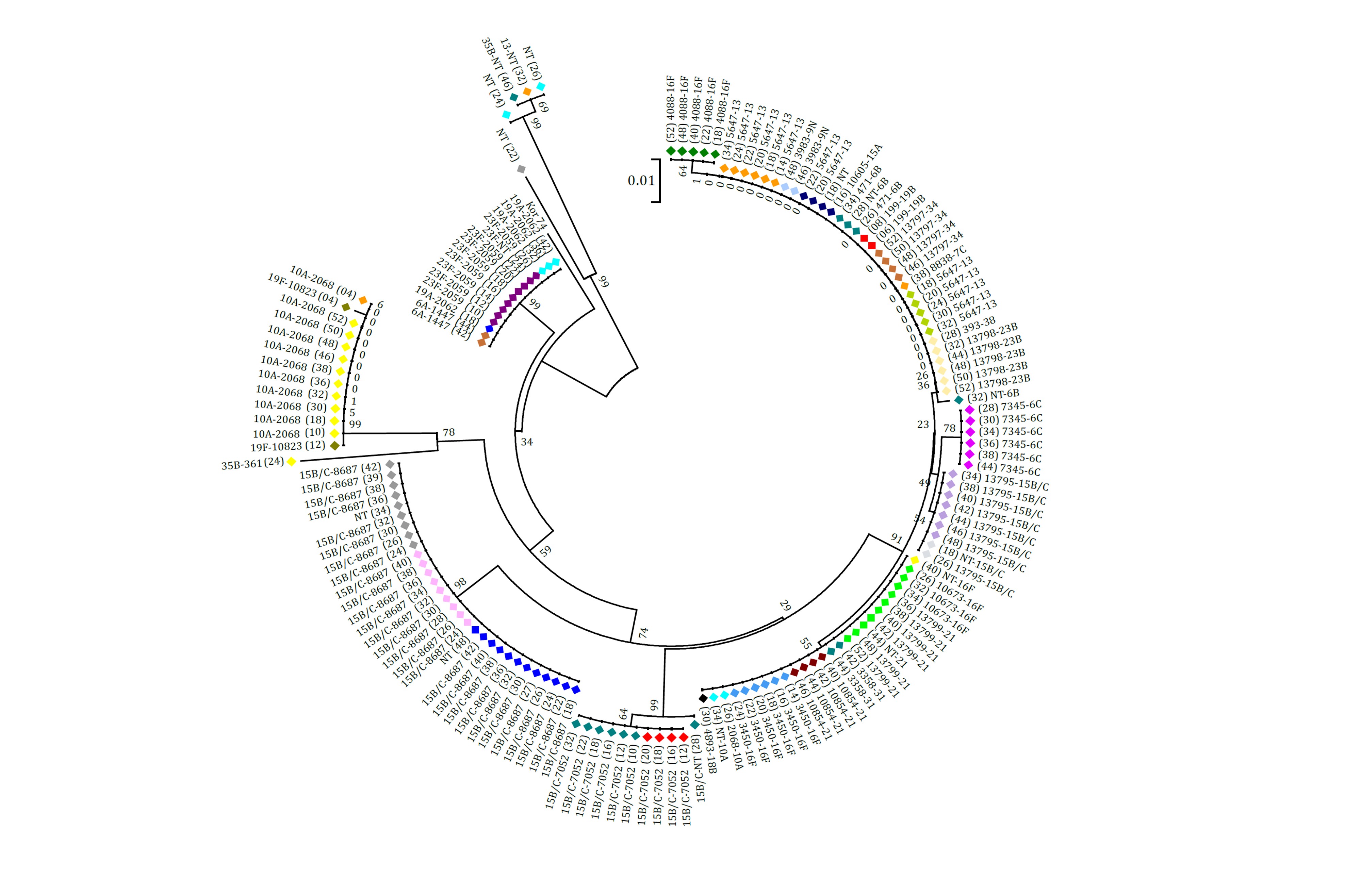


**Figure S3:** Phylogenetic tree, based on the transpeptidase domain of the *pbp*2x gene, constructed by MEGA using the neighbour-joining method. Bootstrap values (percentage) are based on 1,000 replications. The scale bar represents the genetic divergence as calculated by the MEGA software. Same colour represents the longitudinal samples from the same infant. The number in brackets indicate the age in weeks at each time-point. The ST detected in each sample is shown as a number, followed by the associated serotype.


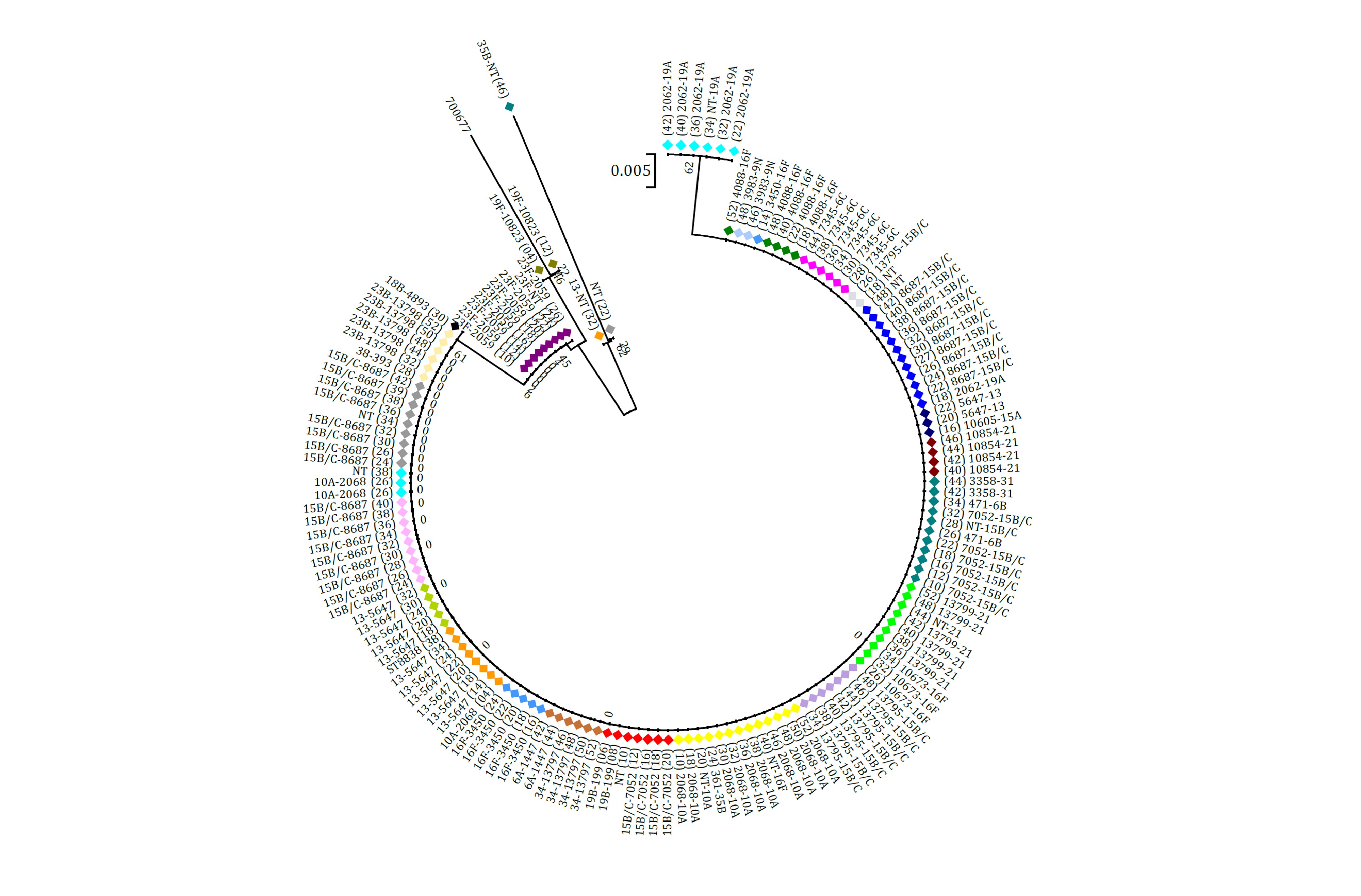


**Figure S4:** Phylogenetic tree, based on the transpeptidase domain of the *pbp*2b gene, constructed by MEGA using the neighbour-joining method. Bootstrap values (percentage) are based on 1,000 replications. The scale bar represents the genetic divergence as calculated by the MEGA software. Same colour represents the longitudinal samples from the same infant. The number in brackets indicate the age in weeks at each time-point. The ST detected in each sample is shown as a number, followed by the associated serotype.
